# Dexamethasone for Severe COVID-19: How Does It Work at Cellular and Molecular Levels?

**DOI:** 10.3390/ijms22136764

**Published:** 2021-06-23

**Authors:** Tomoshige Kino, Irina Burd, James H. Segars

**Affiliations:** 1 Laboratory of Molecular and Genomic Endocrinology, Sidra Medicine, Doha 26999, Qatar; 2 Department of Gynecology and Obstetrics, Johns Hopkins University School of Medicine, Baltimore, MD 21205, USA; iburd@jhmi.edu (I.B.); jsegars2@jhmi.edu (J.H.S.)

**Keywords:** glucocorticoids, glucocorticoid receptor (GR), inflammation, innate immunity, severe acute respiratory syndrome coronavirus-2 (SARS-CoV-2), type I interferons (IFNs)

## Abstract

The coronavirus disease 2019 (COVID-19) caused by infection of the severe respiratory syndrome coronavirus-2 (SARS-CoV-2) significantly impacted human society. Recently, the synthetic pure glucocorticoid dexamethasone was identified as an effective compound for treatment of severe COVID-19. However, glucocorticoids are generally harmful for infectious diseases, such as bacterial sepsis and severe influenza pneumonia, which can develop respiratory failure and systemic inflammation similar to COVID-19. This apparent inconsistency suggests the presence of pathologic mechanism(s) unique to COVID-19 that renders this steroid effective. We review plausible mechanisms and advance the hypothesis that SARS-CoV-2 infection is accompanied by infected cell-specific glucocorticoid insensitivity as reported for some other viruses. This alteration in local glucocorticoid actions interferes with undesired glucocorticoid to facilitate viral replication but does not affect desired anti-inflammatory properties in non-infected organs/tissues. We postulate that the virus coincidentally causes glucocorticoid insensitivity in the process of modulating host cell activities for promoting its replication in infected cells. We explore this tenet focusing on SARS-CoV-2-encoding proteins and potential molecular mechanisms supporting this hypothetical glucocorticoid insensitivity unique to COVID-19 but not characteristic of other life-threatening viral diseases, probably due to a difference in specific virally-encoded molecules and host cell activities modulated by them.

## 1. Introduction

The viruses, intracellular parasites with limited amounts of genetic information, are major threats virtually for all organisms living on earth [1]. They are ancient but emerged as more significant concerns of humankind approximately 20,000–12,000 years ago, when their community density/population greatly increased following the introduction of a new life style “agriculture and pastoralism” [1]. This life style also enabled some viruses maintained in cultivating cattle contaminating into human communities and causing various zoonotic diseases (e.g., variola and influenza) [1]. Although invention of effective vaccinations against many of the pathogenic viruses dramatically reduced their outbreaks and associated diseases during the last century, “new” viruses are still our major threats, occasionally entering human communities [1].

From the beginning of the 21st century, humans encountered several outbreaks of “new” coronavirus [2]. Based on the data of the World Health Organization (WHO), the severe acute respiratory syndrome coronavirus (SARS-CoV) first entered the human community in China during 2003, and affected 8098 people worldwide of whom 774 died (~10% of total incidence) [3]. The less infectious but highly pathogenic middle east respiratory syndrome coronavirus (MERS-CoV) caused its outbreak in 2012 in the Arabian peninsula and subsequently in 2015 in South Korea, and affected a total of 2574 people with 845 associated deaths (~34%) [4]. In December 2019, highly infectious but less pathogenic SARS-CoV-2 invaded a human community at Wuhan, China, possibly from its reservoir animal bats [5,6]. This highly contagious virus rapidly spread to the entire world, and caused a worldwide pandemic of its disease called coronavirus disease 2019 (COVID-19) [7]. COVID-19 is potentially fatal, as it sometimes develops severe pneumonia/respiratory failure and other organ complications, precipitating a total of ~150 million cases and ~3 million deaths (~2%) as of 30 April 2021 [8].

**Figure 1:**
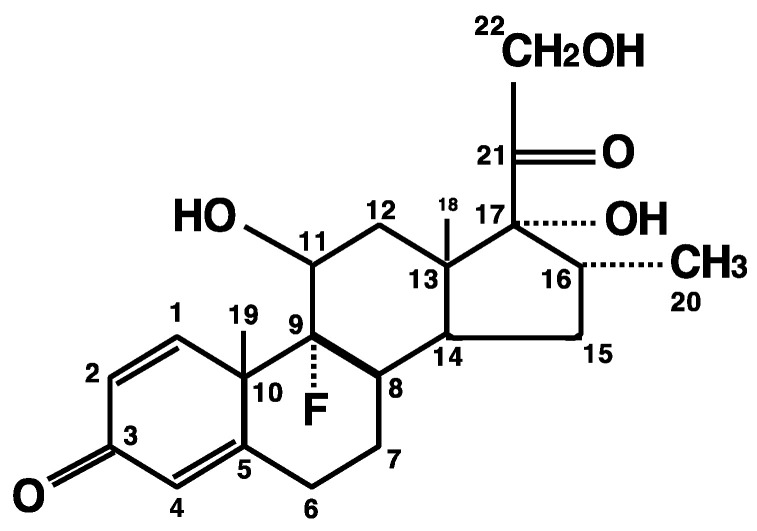
Chemical structure of dexamethasone. Dexamethasone (C_22_H_29_FO_5_: PubChem CID: 5743) is a synthetic pure glucocorticoid fluorinated at carbon-9 with molecular mass of 392.467 g/mol. Similar to cortisol (hydrocortisone), it has a carbonyl oxygen at carbon-3 and a hydroxyethyl at carbon-21 (as carbon-22). Additionally, it harbors a fluoride (F) at carbon-9 and a methyl at carbon-16 (as carbon-20).

Recent successful development of SARS-CoV-2 vaccines provided hope to reduce new infections by SARS-CoV-2, but identification of the effective therapeutic compounds remains an urgent issue for treatment of already ill patients, because it will take considerable time to achieve world-wide herd immunity and there is a possibility that the virus may mutate into a resistant strain before it can be eliminated [9]. Among such candidates, dexamethasone was highlighted as the first effective medicine for severe COVID-19 [10]. This synthetic pure glucocorticoid has ~10-times higher potency and ~3–4 times longer plasma half-life (4–6 h) compared to the endogenous glucocorticoid cortisol (or chemically, hydrocortisone) (Figure 1) [11]. A recent large randomized study employing over 6000 hospitalized cases clearly demonstrated that dexamethasone (oral or intravenous administration) at a dose of 6 mg/day for up to 10 days reduced mortality of the patients requiring mechanical respiratory assistance (thus, severe cases) by one-third, and the mortality of patients requiring O_2_ supplementation without mechanical support (moderate cases) by one-fifth [12]. However, this dexamethasone regimen showed no benefit to mild cases without any respiratory support [12]. These findings were confirmed by several other studies [13,14,15], summarizing that relatively low doses/short use of dexamethasone benefit in severe, but not mild, cases of COVID-19. This suggests that disease severity of COVID-19 as well as timing/amount of the dexamethasone administration are key to a therapeutic benefit of the treatment.

Glucocorticoids induce potent anti-inflammatory/immuno-suppressive effects [16,17]. The endogenous form, cortisol, is the end product of the stress-responsive hypothalamic-pituitary-adrenal (HPA) axis [18,19]. What is curious is that glucocorticoids are only effective for COVID-19 among many other life-threatening infectious diseases, such as bacterial sepsis and influenza pneumonia [20,21,22,23]. In this manuscript, potential therapeutic mechanisms of glucocorticoids unique to COVID-19 are reviewed that might explain this conundrum. Possible mechanisms include infected cell-specific insensitivity to glucocorticoids are discussed in the context of SARS-CoV-2′s viral life cycle inside host cells and the elicited host immune response. We also compare COVID-19 responses to those of other pathogens/viruses for which glucocorticoids show no therapeutic benefit.

## 2. SARS-CoV-2, COVID-19 and Host Immune Response

### 2.1. SARS-CoV-2 and Its Life Cycle inside Host Cells

SARS-CoV-2 is an enveloped, positive-sense single-stranded RNA virus [24]. It forms the *betacoronavirus* genus together with SARS-CoV and MERS-CoV under the diverse family of *Coronaviridae*, which consists of three other genera, *alphacoronavirs*, *gammacoronavirus* and *deltacoronavirus* in addition to *betacoronavirus*. *Alphacoronavirus* and *betacoronavirus* can infect mammalians including humans, whereas *gammacoronavirus* and *detlacoronavirus* have a wider host range including avian species [24]. In humans, *alphacoronaviruses*, such as the human (H) CoV-229E, CoV-NL63, CoV-OC43, and CoV-HKU1 circulate among human communities and develop seasonal respiratory tract infections manifested with common cold-like symptoms [2]. On the other hand, *betacoronaviruses* are highly pathogenic, sometimes developing life-threatening pneumonia requiring O_2_ inhalation and/or mechanical ventilation [2].

To initiate a process of infection to host cells, SARS-CoV-2 binds with its spike (S) protein to host cells first by targeting the heparan sulfate (HS) abundantly presented on the host cell surface and subsequently the angiotensin converting enzyme 2 (ACE2), eventually entering into the cytoplasm of host cells through endocytosis or fusion to the plasma membrane [25]. Pre-processing and subsequent proteolytic digestion of the S protein, respectively by host proprotein convertase furin and host transmembrane protease serine 2 (TMPRSS2), are also required for establishing successful infection [26,27,28]. Therefore, expression of ACE2, furin, and TMPRSS2 on the host cell surface largely determines tropism of SARS-CoV-2 to infecting cells, and influences clinical manifestations/severity of the induced pathologies [29,30]. These molecules are highly expressed on respiratory epithelial cells residing in the upper respiratory tract, whereas pneumocytes II responsible for production and secretion of lung surfactants and residential macrophages/dendritic cells responsible for local immune defense weakly express these molecules [29,30]. Thus, SARS-CoV-2 infection to these cells develop tracheobronchitis as well as bronchopulmonary/organizing pneumonia and associated alveolar damage [31,32,33], mimicking radiographic findings of glucocorticoid-sensitive bronchiolitis obliterans organizing pneumonia (BOOP) but different from those caused by influenza A and other viruses that also target the respiratory tract [34,35,36,37].

**Figure 2:**
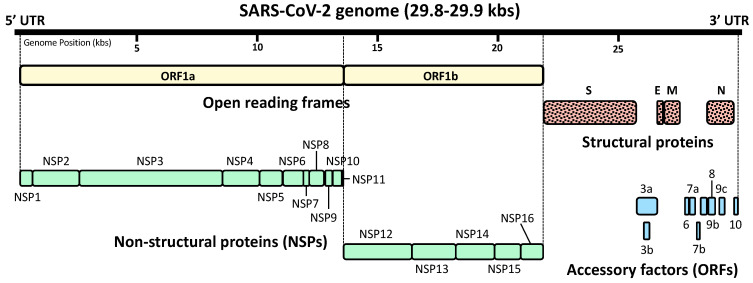
SARS-CoV-2-encoding proteins expressed from its RNA genome. SARS-CoV-2 expresses a total of 29 proteins from its positive-sense single-stranded RNA genome. Open reading frame 1a (ORF1a) and ORF1b are expressed after digestion by the viral protease 16 non-structural proteins (NSPs) in which ORF1a and ORF1b respectively encode 11 proteins (NSP1 to NSP11), and five proteins (NSP12 to NSP16). SARS-CoV-2 also expresses four structural proteins, spike (S), envelop (E), membrane (M), and nucleocapsid (N) proteins, as well as nine accessory factors, ORF3a, 3b, 6, 7a, 7b, 8, 9b, 9c and 10, from their own ORFs. NSP: non-structural protein, ORF: open reading frame, UTR: untranslated region.

After successful infection to host cells, SARS-CoV-2 expresses, from several open reading frames (ORFs) of its genomic RNA, a total of 29 proteins that consist of four structural proteins, sixteen non-structural proteins (NSPs), and nine accessory factors [38,39,40,41] (Figure 2). NSPs are translated either from ORF1a and ORF1b, and are the molecules participating in viral replication, proteasomal digestion of viral polypeptides and modulation of host functions [38,39]. Accessory proteins encoded by their own ORFs also contribute to the modulation of host cell functions by targeting various host molecules [38,39]. Thus, these SARS-CoV-2-specific NSPs and accessory proteins influence its pathogenicity [41,42]. In addition, their high variability—even inside individual coronavirus species—may explain differential pathologic impact observed over different strains of the same species [41,42]. Upon synthesis of these viral proteins, replication of the viral genome is initiated in the host cell cytoplasm first by producing the negative-sense RNAs from the positive-sense viral RNA genome of the infected viral particle [2,43]. These are then used for massive production of the full-length positive-sense viral RNA genomes for translating additional viral proteins as well as packaging into new infectious virions [2,43]. These viral RNAs are N^6^-methyladenosine (m^6^A)-modified at eight sites residing in their 3′ portion [44]. This modification of viral RNAs by the host system appears to be disadvantageous for SARS-CoV-2 because mutations at these sites accumulate in variant strains that emerged after host immune surveillance [44].

Assembly of new viral particles is organized first in the endoplasmic reticulum and then in the Golgi apparatus of the host cells, and new virions created are liberated from the infected cells using host cell machinery of exocytosis [2]. As explained, most of the replication processes of SARS-CoV-2 are carried out inside the host cytoplasm in contrast to the influenza virus whose genomic RNAs are synthesized inside the nucleus and are exported to the cytoplasm for protein translation and packaging into virions [45]. Since replication of SARS-CoV-2 depends on various host biologic systems, its activity influences the clinical picture and disease severity of COVID-19.

### 2.2. COVID-19 and Host Immune Response

**Figure 3:**
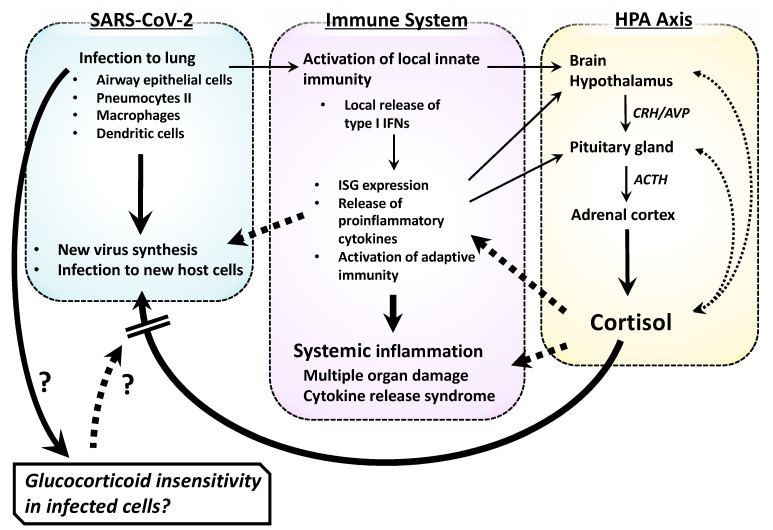
Interaction network between the SARS-CoV-2 infection at local tissues, the host immune system and the HPA axis. SARS-CoV-2 initially infects the lung (e.g., respiratory epithelial cells, pneumocyte II and residential macrophages and dendritic cells) and synthesize its new viral particles inside these cells. Infection of the virus to host tissues activates local innate immune system and production of type I IFNs, which in turn stimulate expression of anti-viral ISGs and release of proinflammatory cytokines, and activate adaptive immunity. Such host immune activation leads to induction of the systemic inflammation, which sometimes progresses into multiple organ damage/cytokine release syndrome. Local inflammation as well as circulating proinflammatory cytokines activate the HPA axis, and stimulate secretion of its end-effector cortisol into systemic circulation. The liberated cortisol strongly suppresses tissue inflammation but also inhibits host anti-viral innate immunity. Coincidentially, the virus appears to block the latter effect of cortisol in infected cells by developing glucocorticoid insensitivity during the process of shifting host cell activities toward its own replication. Solid lines indicate positive effects, while dashed lines are for negative effects. ACTH: adrenocorticotropic hormone, CRH: corticotropin releasing hormone, IFN: interferon, ISG: IFN-stimulated genes.

SARS-CoV-2 causes symptoms of COVID-19 via infection of the respiratory tract in susceptible individuals [2,46] (Figure 3). The clinical picture of COVID-19 is broad, from non-symptomatic or mild upper respiratory tract inflammation to severe bronchopulmonary pneumonia, some cases of which progress into respiratory failure/acute respiratory distress syndrome (ARDS) [47]. In addition, severe COVID-19 develops various complications in other organs, including dysfunctions of the cardiovascular, gastrointestinal, renal, metabolic, reproductive, hemo-coagulation, and central nervous systems [47,48]. Although direct infection of SARS-CoV-2 to vascular endothelial cells and intestinal epithelial cells was reported [49,50], most of the extra-pulmonary manifestations are caused by host immune perturbation represented by cytokine release syndrome [51,52,53]. This syndrome is characterized by massive circulation of various proinflammatory cytokines (e.g., IL-1, IL-2, IL-6, IL-8, IL-12, IL-18 and tumor necrosis factor (TNF) α) released from inflamed lung tissues/immune organs that eventually cause uncontrolled systemic inflammation and associated multi-organ damage/failure [54]. Severe COVID-19 sometimes causes patient death, particularly in the subjects harboring various risk factors, such as male sex, pregnancy, age over 65, obesity, diabetes mellitus, hypertension, and cardiovascular diseases [48,55,56,57]. Although much is not known about their underlying mechanisms, proinflammation associated with some of these factors (e.g., old-age, obesity, diabetes mellitus, and ischemic cardiovascular diseases) appears to increase susceptibility of developing severe COVID-19 [58,59,60].

The innate immune system is a first-line host immune defense against viral infection, by sensing invading viruses with its pattern recognition receptors (PRRs) expressed either on the cell surface or inside the cytoplasm (e.g., toll-like receptors: TLRs, retinoic acid-inducible gene I: RIG-I and the NOD-like receptor family member NOD-, LRR- and pyrin domain-containing 3: NLRP3) [61,62] (Figure 3). Activated PRRs then stimulate the secretion of type I interferons (IFNs) (e.g., IFNα and IFNβ) from infected cells, such as resident pneumocytes, macrophages, and dendritic cells [63]. The locally liberated IFNs bind to their cognate receptors expressed on their responsive cells, activate lines of downstream transcription factors including the interferon regulatory factors (IRFs), signal transducers and activators of transcription (STATs), nuclear factor of κB (NFκB) and activator protein-1 (AP-1), which in turn stimulate the expression of hundreds of IFN-stimulated genes (ISGs) whose encoding proteins harbor various modes of anti-viral activities [64]. These IFNs also enhance the cytolytic activity of CD8^+^ T lymphocytes (T-cells) for clearing the infected cells. Although type I IFNs play major roles, IFNγ (type II IFN) and IFNλ (the type III IFN) also contribute to anti-viral activity of the innate immune system [64]. Furthermore, type I IFNs stimulate local production of a variety of pro-inflammatory cytokines, chemokines, and bioactive molecules, which altogether contribute to the establishment of local and systemic inflammation by increasing vascular permeability, attraction of granulocytes/macrophages, and subsequent activation of adaptive immunity [63,64]. This second-line of immune defense against viral infection is characterized by “memory” of the previous exposure to “non-self” antigens, and is organized mainly by B-cells, and CD4^+^ and CD8^+^ T-cells for production of specific immunoglobulins and induction of memory-guided cytotoxicity [65]. Since SARS-CoV-2-infected patients are virtually naïve to this infection, type I IFN-mediated innate immunity plays critical roles in host protection against this virus [66].

Recent in-depth immune/inflammatory profiling on COVID-19 patients revealed several characteristic changes in their production of cytokines and chemokines, and sub-populations of peripheral lymphocytes. SARS-CoV-2 generally induces robust immune response through activation of both innate and adaptive immunities that results in a massive release of various bioactive molecules into circulation [67,68]. Among them, most of the cytokines associated with cytokine release syndrome, such as IFNα, IL-1α/1β, IL-6, IL-10, IL-18, and TNFα, have serum concentrations correlated with disease severity, whereas patients with life-threatening COVID-19 demonstrate an additional elevation of other inflammatory factors, including IL-16, IL-21, IL-23, IL-33, IFNγ, C-C motif chemokine ligand (CCL) 11, CCL26, and thrombopoietin [67]. Such broad changes in circulating inflammatory factors are also corroborated with elevation of the inflammatory markers, ferritin, and C-reactive protein (CRP), as well as significant lymphopenia due to the reduction of circulating CD4^+^ and CD8^+^ T-cells [56,67]. Of note, patients with severe COVID-19 demonstrate progressive elevation of circulating IFNα and viral load [67]. A fraction of severe COVID-19 cases harbors inactivating mutations in the genes functional in type I IFN-mediated innate immunity or have in their circulation auto-antibodies that bind to and neutralize type I IFNs [69,70,71,72]. In addition, these patients demonstrate aberrant elevation of the cytokines functional against fungal and/or parasite infections [67]. Thus, these pieces of evidence strongly suggest that an insufficient host type I IFN-mediated anti-viral immunity and subsequent failure in eradicating SARS-CoV-2 infection are key for progressing into severe COVID-19. Failure to diminish viral invasion by innate immunity leads to uncontrolled production of a variety of inflammatory factors characterized as cytokine release syndrome (Figure 3). In line with this possibility, a recent study employing an animal model of SARS-CoV-2 infection, together with actual patient samples, indicated the reduced innate antiviral defense coupled with exuberant production of pro-inflammatory cytokines is a defining feature of severe COVID-19 [57]. However, cytokine release syndrome is not specific to COVID-19 but can be observed in various infectious/inflammatory diseases, including avian influenza pneumonia, bacterial sepsis, anti-cancer chimeric antigen receptor T cells (CAR-T) therapy, and graft-versus-host reaction after organ transplantation, indicating that this syndrome is an ultimate consequence of uncontrolled systemic inflammation regardless of pathologic causes/processes [54,73].

**Figure 4:**
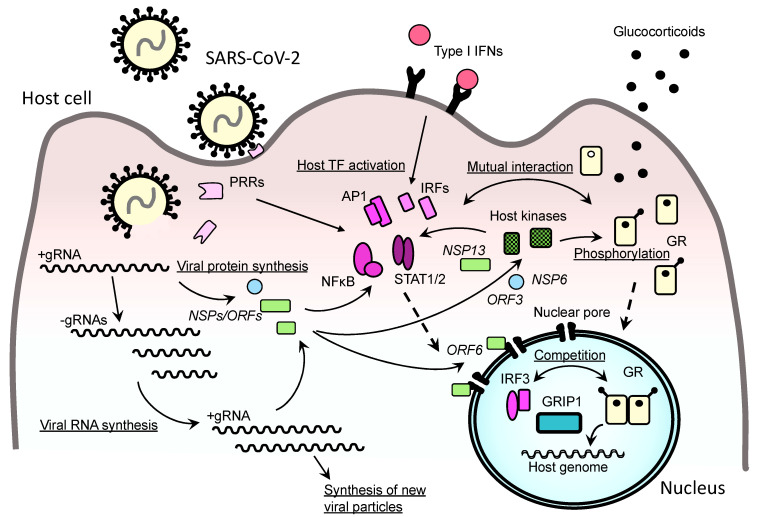
SARS-CoV-2′s life cycle in a host cell and its expected interference with the GR signaling pathway. Upon infection to a host cell, SARS-CoV-2 releases its positive-sense single-stranded genomic (g) RNA into the cytoplasm and initiates synthesis of its RNAs and their encoding proteins. On the other hand, the infection activates local anti-viral innate immune system through host PRRs, which in turn stimulate production/release of type I IFNs. Activated PRRs and released IFNs further stimulate host transcription factors (e.g., NFκB, AP1, STATs and IRFs) for the expression of ISGs and proinflammatory cytokine genes. Some of the produced viral proteins (e.g., NSP6, NSP13, ORF3, and ORF6) shift host cell activities toward viral replication, such as by changing the activities of host kinases, transcription factors, nuclear pore complex, and RNA processing machinery, whereas they downregulate host innate immunity. In the process of modulating host cell activities, SARS-CoV-2 appears to suppress coincidentally the facilitative effects of GR on viral replication by affecting multiple components of its intracellular signaling pathway. Host and viral proteins are indicated with regular and italic letters, respectively. AP1: activator protein-1, GR: glucocorticoid receptor, GRIP1: glucocorticoid receptor-interacting protein-1, IFN: interferon, IRF: interferon regulatory factor, ISG: IFN-stimulated gene, NFκB: nuclear factor of κB, NSP: non-structural protein, ORF: open reading frame, PRR: pattern recognition receptor, STAT: signal transducer and activator of transcription.

At a molecular level, the reduced type I IFN-mediated innate immunity observed in severe COVID-19 may be through affecting multiple components of this defense system with SARS-CoV-2-encoded molecules [39] (Figure 4). In a transient transfection-based screening in human 293T cells, overexpression of NSP1, NSP3, NSP12, NSP13, NSP14, ORF3, ORF6, or M protein inhibits Sendai virus-induced activation of the IFNβ promoter [74]. Specifically, NSP6 binds TANK-binding kinase 1 (TBK1) and suppresses phosphorylation of IRF3, whereas NSP13 binds and blocks TBK1 phosphorylation and inhibits its kinase activity [75]. ORF6 binds to the importin karyopherin α2 (KPNA2), an essential component of the host cytoplasmic-to-nuclear transport system residing in the nuclear pore, and inhibits nuclear translocation of IRF3 [75]. Furthermore, two sets of the viral proteins including NSP1, NSP6, NSP13, ORF3a, ORF3b ORF7a, ORF7b, and/or M protein antagonize to type I IFN signaling by blocking the phosphorylation of STAT1 and STAT2, a process required for activating these transcription factors to stimulate ISG transcription. In addition, ORF6 interferes with nuclear translocation of these transcription factors through the nuclear pore, additionally contributing to its inhibition of ISG expression [75,76].

## 3. HPA Axis and Its End Effectors Glucocorticoids in Controlling Inflammation

### 3.1. HPA Axis and Endogenous Glucocorticoids

The HPA axis governs adaptive responses against any unforeseen stressful stimuli including viral infection and associated tissue inflammation [18] (Figure 3). It senses tissue inflammation through neural and humoral pathways. For example, inflammatory signals generated in local inflamed tissues are first sensed by peripheral neurons and are transferred to the brain hypothalamic paraventricular nucleus, a central component of the HPA axis, through the afferent autonomic neural pathways [18,19,22]. On the other hand, proinflammatory cytokines (e.g., IL-1, IL-6 and TNFα,) secreted from local inflammatory sites reach the hypothalamic nucleus and/or the anterior pituitary gland of the HPA axis, and stimulate directly or indirectly the secretion of their signaling molecules, corticotropin-stimulating hormone (CRH)/arginine vasopressin (AVP) from the hypothalamus and the adrenocorticotropic hormone (ACTH) from the pituitary gland [18,19,22]. Secreted ACTH then stimulates the adrenal gland cortex for their production/secretion of the end effector hormone cortisol [19]. This steroid reaches every component of the human body through systemic circulation and alters its biological activities via an intracellular receptor molecule, the glucocorticoid receptor (GR) [77]. Cortisol-free GR resides in the cytoplasm, and upon binding this steroid, translocates into the nucleus through nuclear pores by communicating with the nuclear pore complex including importins [78,79]. Nuclear GR alters transcription rates of glucocorticoid-responsive genes positively or negatively through binding to its DNA recognition sequences called glucocorticoid response elements (GREs) located in the regulatory elements of these genes, or through indirect association to regulatory elements via protein–protein interaction with other transcription factors/cofactors already attracted to them [80]. The DNA-associated GR then accumulates at its residing regulatory elements the transcriptional machinery including the RNA polymerase II and various cofactor molecules, such as p300- and p160-type histone acetyltransferase coactivators [80]. GR also negatively regulates transcription of some glucocorticoid-responsive genes by associating with negative GREs including the inverted repeat negative GREs and by attracting repressive cofactors and/or disrupting the optimal transcriptional condition established by other transcription factors [81,82]. Transcriptional effects of GR are tissue-/cell type-specific, owing mainly to the epigenetic modulation of chromatin DNA/proteins, such as DNA methylation and chemical modification (e.g., methylation, acetylation, and phosphorylation) of chromatin-associated histones, which alters the accessibility of GR to their harboring genome DNA [80]. In addition to such direct/indirect transcriptional regulation known as genomic effects, GR influences various cellular activities known as its non-genomic effects by interacting with their key molecules through direct/indirect protein–protein interactions [83]. For example, GR facilitates mRNA degradation in a ligand-dependent fashion by communicating with hundreds of mRNAs and by activating the GR-mediated mRNA decay system that consists of several molecules including those functional in the m^6^A-mediated RNA decay [84,85,86]. Through these distinct biologic actions of GR, cortisol ultimately exerts its anti-stress effects by inducing numerous qualitative and/or quantitative changes in organs/tissues, such as of the immune, metabolic, neural, and cardiovascular systems [19,87]. However, the changes induced by the HPA axis/cortisol are transient, as stimulation of the HPA axis is promptly reset by the intrinsic negative feedback loop consisting of direct suppression of its upper regulatory centers by secreted cortisol [18] (Figure 3). If this negative feedback loop fails to function properly, for example by exposure to repetitive stressful stimuli, lines of adverse effects associated with prolonged and/or elevated secretion of cortisol may emerge, such as increased susceptibility to pathogen infection, mental/emotional changes (e.g., psychosis and depression), hyperglycemia, osteoporosis, and hypertension [19,87]. As part of the negative feedback loop, the cortisol massively secreted in response to inflammation strongly suppresses the immune system and associated inflammatory reactions, helping to curtail organ damage elicited by highly activated and/or prolonged tissue inflammation [87]. On the other hand, unstressed levels of cortisol are essential for the maintenance of proper immune functions, such as of innate immunity that mediates protection against pathogen infection [77,88].

### 3.2. Glucocorticoid Effects on Immune System

Pharmacologic or stress-equivalent doses of glucocorticoids induce diverse and intense suppressive effects on the overall immune system [16,18,89]. Since their actions are immune component-/cell type-specific, their observed effects depend on the original pathologic causes and/or associated processes of the immune activation [16,89]. In general, pharmacologic doses of glucocorticoids exert their anti-inflammatory effects by affecting both innate and adaptive immunity through targeting the following three components: (1) production of bioactive molecules, (2) migration of macrophages/leukocytes into local inflamed sites, and (3) regulation of cellular immunity (T helper (Th) 1- and Th17-mediated) and humoral immunity (Th2-mediated) [89]. For example, inflamed tissues and residing immune cells produce many bioactive molecules, such as chemokines, prostaglandins, histamine, bradykinin, and nitric oxide for increasing vascular permeability and subsequent attraction of circulating leukocytes/macrophages to local inflammatory sites, and glucocorticoids regulate (suppress in most cases) production/expression of most of these molecules [90,91,92,93]. In addition, glucocorticoids strongly suppress production of many proinflammatory cytokines, such as IL-1, IL-2, IL-6, IL-12, and IL-17, at inflamed sites [94,95,96,97,98]. Furthermore, glucocorticoids increase the numbers of circulating neutrophils but reduce eosinophils and basophils [89]. Glucocorticoids also enhance clearance of foreign antigens, toxins, micro-organisms, and dead cells from inflamed sites by enhancing opsonization of the scavenger systems and by stimulating phagocytosis of macrophages [99]. Glucocorticoids suppress cellular immunity but stimulate humoral immunity by changing differentiation of CD4^+^ T-cells and B-cells through modulating production of responsible cytokines as well as antigen presentation of dendritic cells to T-cells [100].

### 3.3. Glucocorticoid Effects on Type I IFN-Mediated Innate Immunity

In general, glucocorticoids suppress type I IFN-mediated innate immunity by inhibiting their intracellular signaling pathways and subsequent expression of ISGs [101,102] (Figure 3). This is in part through NFκB and AP-1, which are well known transcription factors whose activity is repressed by GR via direct protein-protein interactions [103] (Figure 4). Glucocorticoids also inhibit TBK1 for its phosphorylation of IRF3 and IRF7, and their subsequent induction of IFNβ and ISGs [104,105]. Furthermore, GR antagonizes to the transcriptional activity of IRF3 by completing the glucocorticoid receptor-interacting protein 1 (GRIP1, or steroid receptor coactivator 2: SRC2) because both IRF3 and GR employ as their coactivator this large platform of the p160 family protein in their transcriptional regulation [106]. IRF3 also employs as its coactivator a p65 component of the NFκB transcription factor for IRF-stimulated response element (IRSE)-driven ISGs, whereas GR sequesters this p65 from IRF3 and inhibits expression of these ISGs [107].

## 4. Insensitivity to Glucocorticoids in SARS-CoV-2-Infected Cells: A Key Mechanism Underlying the Beneficial Effects of Glucocorticoids on Severe COVID-19?

For treating any viral diseases causing systemic inflammation and subsequent organ/tissue damage, the following two strategies are regularly employed: (1) reducing viral entry/replication by targeting key components of these viral activities; and (2) suppressing virus-induced inflammation and subsequent organ damage by intervening in relevant host immune pathways [108]. For example, various compounds that block entry of the virus to host cells (e.g., entry receptor competitors and host protease inhibitors) as well as those inhibiting viral genome synthesis (e.g., nucleotide analogues) have been successfully developed or are under intense investigation for many pathogenic viruses including SARS-CoV-2 [24,109,110,111]. Glucocorticoids are representatives of the latter approach, suppressing organ inflammation by interfering with various components of the host immune system as discussed above (Figure 2). However, blocking inflammation/immune system by glucocorticoids also limits host ability to impede viral invasion, thus this property of glucocorticoids increases the chance of viral replication and subsequent worsening of elicited diseases [102,112,113]. After long debates with numerous clinical trials, glucocorticoids turned out to be ineffective or even harmful for many infectious diseases including bacterial sepsis and severe influenza pneumonia, which may develop into ARDS and cytokine release syndrome similar to COVID-19 [73,114]. Furthermore, patients under glucocorticoid treatment are susceptible to the viruses causing opportunistic infections, including adenovirus, cytomegalovirus, and herpes simplex virus [115]. Thus, it is likely that glucocorticoids exert their beneficial effects on COVID-19, not just by controlling organ inflammation, but due to additional therapeutic properties unique to SARS-CoV-2 infection that may influence anti-viral immunity. The apparent conflict observed between the known adverse actions of glucocorticoids on the innate immunity that could allow SARS-CoV-2 replication/invasion and their beneficial effects suppressing SARS-CoV-2-elicited inflammation suggests the hypothesis that the former adverse effects of glucocorticoids might be opposed by glucocorticoid insensitivity (or resistance) that develops in virally infected cells (Figure 3). Notably, a similar state specific to infected cells has been reported in other viral infections [116,117]. Furthermore, pathology-associated insensitivity to therapeutic glucocorticoids is a significant concern for several autoimmune/allergic diseases including rheumatoid arthritis, systemic lupus erythematosus, and bronchial asthma, whereas local glucocorticoid sensitivity physiologically fluctuates along circadian rhythms [19,111,118,119]. Thus, SARS-CoV-2 appears to develop coincidental glucocorticoid insensitivity in virally infected cells, possibly in a process of activating the immune system/inflammation as well as modulating the innate immune system and/or shifting cellular functions towards its replication. For example, SARS-CoV-2 infection stimulates host immune response by activating NFκB and AP-1, whereas these transcription factors repress GR activity through physical interaction to this receptor, as evident in rhinovirus infection where activation of these transcription factors causes glucocorticoid insensitivity in virus-infected airway epithelial cells [110,117] (Figure 4). IFN-downstream factors, IRF3 and IRF9 activated upon SARS-CoV-2 infection, shares with GR the GRIP1 coactivator for supporting their transcriptional activity [106,107,120]. This plausible mechanism raises the possibility that these transcription factors compete with GR in this molecular complex, resulting in repression of the latter’s transcriptional activity. In addition, SARS-CoV-2 infection inhibits importin KPNA2 with its ORF6 and suppresses nuclear translocation of IRF3 for downregulating IFN-mediated ISG expression [75]. ORF6 also interacts with the NUP98-RAE complex of the nuclear pore complex, and inhibits the latter’s activity for bi-directional transport of RNA-binding ribonucleoproteins through the nuclear pore [121] (Figure 4). Since GR also employs these nuclear import/export systems for its intracellular shuttling [122], SARS-CoV-2 may alter intracellular localization of GR and eventually disturb its functions through ORF6.

In addition to targeting these transcription factors and the nuclear pore-associated transport system, SARS-CoV-2 globally alters phosphorylation profiles of over 40 host and viral proteins by modulating the activity of ~100 host kinases [38,123] (Figure 4). The kinases activated by SARS-CoV-2 infection include those involved in the p38/mitogen-activated protein kinase (MAPK)-mediated signaling (e.g., p38 MAPK, MAP2K3/4/6, and MAPKAPK2/3), whereas downregulated kinases are functional, such as in the organization of cell growth, cell cycle, and cytoskeleton architectures [123]. The p38 MAPK pathway is involved in the activation of NFκB and AP-1, which can repress GR transcriptional activity as discussed above. This kinase also phosphorylates GR and modulates its transcriptional activity [124,125]. Suppression of cyclin-dependent kinase (CDK) 2 activity by SARS-CoV-2 causes G2/M arrest in the cell cycle of infected cells for supporting productive infection [123]; whereas GR transcriptional activity is downregulated in G2/M-arrested cells [126]. Besides these kinases, SARS-CoV-2 stimulates the casein kinase II (CKII), CDK5, RAC-α serine/threonine-protein kinase (AKT1), and glycogen synthase kinase 3β (GSK3B) [123]. These kinases can phosphorylate GR and/or its regulatory factors including the CREB-regulated transcription coactivator 2 (CRTC2), histone deacetylase 2 (HDAC2) and sirtuin 1 (Sirt1), and suppress GR activities inside the infected cells [127,128,129,130,131].

## 5. Other Potential Mechanisms Supporting the Beneficial Effects of Glucocorticoids on COVID-19

### 5.1. Insufficient Activation of the HPA Axis in Severe COVID-19

It is clear that proper activation of the HPA axis and subsequent secretion of cortisol is critical for survival against bacterial sepsis/shock and potentially fatal viral infections [132,133,134]. For example, enlargement of adrenal glands is identified in the subjects who survived in septic shock, whereas its absence is associated with mortality of the patients [135]. Relative adrenal insufficiency is also reported in patients with meningococcal sepsis [136]. In a murine model of influenza virus and bacterial (*Listeria monocytogenes*) co-infection, stimulation of the HPA axis/secretion of corticosterone (an endogenous glucocorticoid in rodents) upon the viral infection is necessary for controlling the inflammatory response and preventing lethal immunopathology caused by a secondary bacterial infection [134]. Thus, insufficient activation of the HPA axis/cortisol secretion could also occur in the patients with severe COVID-19, and reduced production of cortisol might precipitate severe cases or death. In agreement with a possibility of glucocorticoid insufficiency in severe COVID-19, “short” administration of relatively “low” dose dexamethasone would supplement insufficient cortisol secretion from the adrenal glands in severely ill subjects and help with their survival, although no evidence supporting this speculation has been reported. The biologic mechanism(s) underlying the observed beneficial effects of secreted cortisol/corticosterone upon these pathogen infections is (are) not fully understood [133], but a fine balancing between immunosuppressive effects of secreted cortisol/administered glucocorticoids and anti-pathogenic activity as a function of a maintenance of immune homeostasis appears to be involved.

### 5.2. Acceleration of Lung Regeneration by Glucocorticoids

Glucocorticoids are well-known factors critical for fetal lung maturation by promoting the production of lung surfactants from alveolar pneumocytes II [137,138,139,140,141]. Thus, they are used antenatally in pregnancies at risk of preterm birth to avoid the development of respiratory distress syndrome (IRDS) in premature infants [142,143]. In addition to the effect on lung surfactants, glucocorticoids exert diverse actions to facilitate fetal lung maturation [144]. For example, glucocorticoids stimulate differentiation of alveolar progenitor cells into pneumocytes I and II, suppress proliferation of alveolar mesenchymal cells for inducing mesenchymal thinning, and regulate the production of collagen and elastin fibers in the alveolar mesenchyme to maintain its proper elasticity [145,146,147,148,149]. Some of these glucocorticoid actions associated with fetal lung maturation conducted as part of its developmental processes may also be functional in regeneration of the lung damaged by COVID-19 pneumonia, such as assisting in the inflation of collapsed alveoli, thinning alveolar walls and/or suppressing mesenchymal fibrosis. Furthermore, pneumocytes II, direct targets of SARS-CoV-2 infection, are key cells for maintaining alveolar fluid balance, fibrinolysis, and defense against pathogen invasion by acting as immunomodulatory cells [150]. They also contribute to repairing the alveolar epithelial layer by differentiating into pneumocytes I and by phagocyting apoptotic pneumocytes II [150]. Thus, tropism of SARS-CoV-2 to pneumocytes II through ACE2 might influence pathology of COVID-19 pneumonia and underlie the beneficial effects of glucocorticoids, in contrast with other viruses (e.g., influenza A) that do not employ this entry receptor and are not responsive to these steroids.

### 5.3. lncRNAs and N^6^A Modification of RNAs

In addition to modulating protein molecule-mediated cellular systems, SARS-CoV-2 might alter GR actions through changing the activities/expression of the host long non-coding (lnc) RNAs. LncRNAs are not translated to proteins but are intrinsically functional, primarily in the transcriptional regulation of protein-coding genes [151]. Some lncRNAs are known to influence GR transcriptional activity through physical interaction with this receptor (e.g., growth arrest-specific 5: Gas5 and steroid RNA coactivator: SRA) [152,153]. SARS-CoV-2 skews host mRNA processing by targeting multiple components of this system, such as suppression of the transcription elongation of mRNAs by affecting the elongation factors associated with host RNA polymerase II as well as blocking nuclear to cytoplasmic translocation of mRNA-binding ribonucleoproteins by affecting the nuclear pore complex [2,39]. These actions of SARS-CoV-2 on host mRNA processing are likely to influence production and/or subcellular localization of host lncRNAs as well, further modulating their actions on GR in infected cells.

It is also possible that N^6^A modification of RNAs contributes to the beneficial effects of glucocorticoids on severe COVID-19. N^6^A modification influences various properties of RNAs, such as their subcellular localization, efficiency of translation into encoding proteins and fate (e.g., degradation/decay) [154,155,156]. N^6^A modification negatively regulates SARS-CoV-2 replication, while it facilitates propagation of some other viruses including influenza A virus [44,157]. It also affects host immune activity by changing the expression of immune-related molecules whose encoding mRNAs harbor this chemical modification [156]. Furthermore, chronic stress/exogenous glucocorticoids alter N^6^A modification on mRNAs in mice brains and change their stress response [158]. Thus, it is possible that glucocorticoids alter functions and/or fate of SARS-CoV-2 RNAs and/or host immune response against this virus through altering N^6^A modification of viral RNAs, host mRNAs, and/or lncRNAs in a way specific to this virus. Although pathophysiologic roles of lncRNAs and RNA N^6^A modification in COVID-19 have not been fully examined, future studies may reveal their important roles in COVID-19 pathologies, particularly their influence on glucocorticoid actions in infected cells.

### 5.4. Bacterial Co-Infection

COVID-19 pneumonia is associated with low incidence of bacterial co-infection (~4% of all cases) compared to influenza pneumonia whose co-infection rates reach ~30% in hospitalized patients [159]. Although influence of bacterial co-infection to the outcome of glucocorticoid treatment in COVID-19 is not known, glucocorticoids generally worsen bacterial diseases [115]. Thus, this feature of COVID-19 may additionally contribute to the beneficial effects of glucocorticoids on severe COVID-19 pneumonia, not like influenza pneumonia for which these steroids are even harmful in part through worsening pathologies caused by co-infected bacteria [21,160].

## 6. Conclusive Remarks and Future Perspectives

After long failure of glucocorticoids in the treatment of systemic inflammation caused by infectious diseases, COVID-19 caused by SARS-CoV-2 has been identified as a pathology for which dexamethasone is beneficial [12,13,14,15]. The therapeutic failure of glucocorticoids in most infectious diseases is apparently associated with their adverse effects of allowing pathogen invasion/replication, although they are quite effective for suppressing the induced inflammation. Thus, SARS-CoV-2 infection might harbor unique features that make glucocorticoids an effective treatment. The life cycle of SARS-CoV-2 and the intracellular glucocorticoid signaling system are highly interconnected with one another as has been discussed. SARS-CoV-2 infection develops significant and global changes in infected cells, suppressing innate immunity, altering their growth, arresting the cell cycle at the G2/M check-point and changing cytoskeletal architectures, through modulating the activity of various host kinases, RNA processing machinery, and cytoplasmic-nuclear trafficking, many of which influence GR actions inside the infected cells [2,39,78,125]. SARS-CoV-2 induces these changes in host cells primarily for promoting its replication, but would accidentally modulate the intracellular actions of GR and contribute to the therapeutic effects of glucocorticoids on severe COVID-19. Importantly, this unintended potential influence of SARS-CoV-2 on the glucocorticoid signaling system appears to be multifactorial. Direct modulation of GR actions through physical interaction of SARS-CoV-2 proteins to this receptor or its transcriptional cofactors is possible as reported for other viruses [161,162,163], but recent findings obtained in the analysis on an interaction network between host and viral proteins do not support this possibility [38].

As a clinical perspective, polypharmacy employing glucocorticoids and other therapeutic compounds appears to be a realistic future of glucocorticoid-mediated COVID-19 therapy, similar to various autoimmune, allergic and/or lymphoproliferative diseases for which glucocorticoids are used as part of therapeutic regimens also including several other compounds [11]. In agreement with this approach, combinatory use of IFNα along with dexamethasone synergistically shortens hospital stay and improves clinical symptoms of hospitalized COVID-19 patients [164]. IL-6 receptor antagonists are also promising candidates. A recent clinical trial testing these compounds (tocilizumab and sarilumab) by employing COVID-19 patients, many of whom were also treated with dexamethasone, significantly improved their survival, whereas another study on tocilizumab conducted earlier than this study and not including subjects under dexamethasone treatment failed to show a beneficial effect [46,108,165]. Further intensive research for identifying other effective compounds as well as subsequent establishment of potent multi-drug regimens including glucocorticoids will warrant future treatment for COVID-19.
